# Sedation with volatile anesthetics in the intensive care unit: a new option with old agents

**DOI:** 10.5935/2965-2774.20230394-en

**Published:** 2023

**Authors:** Fernando José da Silva Ramos, Mauricio Henrique Claro dos Santos, Laerte Pastore Junior

**Affiliations:** 1 Department of Anesthesiology, Pain and Intensive Care Medicine, Universidade Federal de São Paulo - São Paulo (SP), Brazil; 2 Adult Intensive Care Unit, Hospital Sírio-Libanês - São Paulo (SP), Brazil

## INTRODUCTION

Since December 2019, when the first cases were described in China, the coronavirus disease 2019 (COVID-19) pandemic has impacted health systems around the world. A significant number of patients have the severe form of the disease, requiring admission to the intensive care unit (ICU).^(1)^ The shortage of beds, equipment and drugs represented an even greater challenge in the management of these patients. The improvised use of operating rooms, which served as ICU beds, and the use of anesthesia equipment for sedation and mechanical ventilation have been described and were employed as heroic measures in the management of these patients.^(2,3,4)^ In this context, the use of volatile anesthetics (VAs) has reappeared as an option for the sedation of critically ill patients.^(3)^

The use of VAs in the ICU has been described for more than 2 decades and is mainly used in Europe and Canada;^(5)^ however, the equipment to administer VAs was only recently approved for use in Brazil. The main VAs used as sedatives in the ICU are sevoflurane and isoflurane. The development of equipment with compact vaporizers adapted for mechanical ventilators in ICUs made it possible to use these agents as an option for sedation. Among the main advantages of using VAs rather than opioids in critically ill patients are earlier awakening, lower use of opioids and shorter time on mechanical ventilation. Other reported benefits of VAs are bronchodilator effects and improved oxygenation, especially in patients with acute respiratory distress syndrome (ARDS). Among the contraindications and limitations of VAs are a personal or family history of malignant hyperthermia, suspected or confirmed intracranial hypertension, severe hemodynamic instability and significant pulmonary secretion with the need for frequent aspiration due to the risk of system obstruction.^(5)^

Three meta-analyses showed that compared to venous sedation, the use of VAs in the ICU resulted in faster awakening and extubation times.^(6,7,8)^ More recently, Meiser et al., in a multicenter noninferiority study of isoflurane compared to propofol, showed that isoflurane was an effective and safe option. Additionally, in the isoflurane group, opioid consumption was lower.^(9)^

Experimental studies have shown that sevoflurane has the ability to reduce lung inflammation in ARDS models.^(10,11)^ Jabaudon et al., in a randomized study, demonstrated that compared with midazolam, the use of sevoflurane in patients with ARDS for a period of 48 hours was related to improved oxygenation and reduced markers of lung epithelial lesions.^(12)^

The use of VAs in the ICU has been more frequently reported in populations of surgical patients. Although there are no contraindications for VAs use in other populations of critically ill patients (e.g., patients with sepsis), further studies are needed.

### How can volatile agents be used in intensive care units?

**Figure 1:**
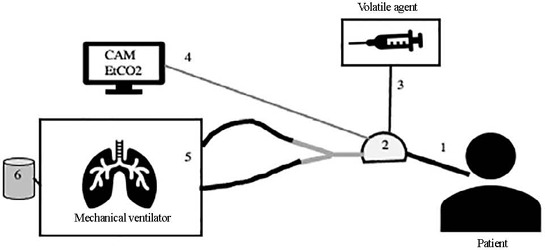
Example of the assembly and arrangement of sedation equipment with volatile agents in the intensive care unit

Volatile anesthetics are an option for sedation in critically ill patients. Table 1 presents the main indications and options for VAs use in the ICU. An example of the assembly of the sedation device for the delivery of volatile agents is presented in figure 1. A humidifier and antibacterial filter are attached to the vaporizer. Although the equipment is easy to assemble and use, the need to acquire specific equipment may be a limitation to the use of VAs. Cost-effectiveness evaluation studies on VAs use in the ICU are still needed. An advantage of using these VAs is the clearance of the drug through pulmonary expiration and a systemic metabolism rate lower than 0.2% for isoflurane and close to 5% for sevoflurane.^(5)^

**Table 1:** Potential use of sedation with volatile agents in the intensive care unit

Potential indications for the use of volatile agents in critically ill patients
1. Prediction of early awakening and short sedation time (Example: postoperative period)
2. Complex sedation requiring multiple agents (propofol-sparing agents or benzodiazepines)
3. Severe bronchospasm
4. Super-refractory status epilepticus

(1) Orotracheal cannula; (2) sedation device containing volatile anesthetic; (3) infusion pump with volatile agent and medication line; (4) gas monitor; (5) mechanical ventilator; (6) activated carbon filter. The volatile agent (3) is infused through an infusion pump to the conservation device and vaporizer (2). Line 4 is the connection from the vaporizer device to the gas monitor. The sedation device (2) should be placed between the orotracheal tube (1) and the ventilator circuit (5). An additional humidification filter should not be fitted. The device recycles expired air. An activated carbon filter must be placed at the outflow of the mechanical fan.

Regarding the occupational safety of VAs in ICU environments and mechanical ventilators, several studies have evaluated the environmental contamination risk and occupational risk of this equipment, demonstrating safe use under certain conditions and with the appropriate equipment.^(13,14,15)^

In conclusion, the use of sevoflurane and isoflurane and inhaled sedation equipment developed for the ICU seems to be an option for specific groups of critically ill patients.
